# Association between an increase in blood urea nitrogen at 24 h and worse outcomes in COVID-19 pneumonia

**DOI:** 10.1080/0886022X.2021.1879855

**Published:** 2021-02-15

**Authors:** Bo Ye, Hongbin Deng, Hanwei Zhao, Jingjing Liang, Lu Ke, Weiqin Li

**Affiliations:** a Department of Critical Care Medicine, Jinling Hospital, Nanjing University School of Medicine, Nanjing, People’s Republic of China; b Department of Critical Care Medicine, Huoshenshan Hospital, Wuhan, People’s Republic of China; c Department of Critical Care Medicine, 902 Hospital of PLA, Bengbu, People’s Republic of China ;; d Department of Endocrinology, Jinling Hospital, Nanjing University School of Medicine, Nanjing, People’s Republic of China; e National Institute of Healthcare Date Science at Nanjing University, Nanjing, China

Since March 2020, coronavirus disease 2019 (COVID-19) has been declared a pandemic, which has been on escalation. Blood urea nitrogen (BUN) has been considered to be an independent risk factor for mortality in COVID-19 patients [1]. However, there is no study assessing the relationship of dynamic change in BUN and worse outcomes in COVID-19.

In this study, we included COVID-19 patients admitted to the Huoshenshan hospital from 5 Feb to 5 March in 2020, which is a newly established, referral, portable hospital for the outbreak of COVID-19 in Wuhan. And then we investigate the relationship between dynamic change in BUN and key clinical outcomes like transfer to intensive care unit (ICU), invasive mechanical ventilation, acute kidney injury (AKI), and in-hospital mortality.

Patients who had BUN level tested on admission and on the second day were considered eligible. Patients who had a known history of chronic kidney disease were excluded. Delta BUN was defined as the change of BUN from admission to 24 h after admission. The study population was divided into two groups according to their delta BUN during the first 24 h (Increase group and Non-increase group). On admission, the epidemiological data, comorbidities, vital signs, and laboratory examination of the study cohort were obtained.

There were 266 patients included for analysis. The mean delta BUN was 1.0 mg/dL (SD 9.5 mg/dL), with 206 patients (77.4%) experiencing a decrease or no change in BUN and 60 patients (22.6%) experiencing an increase. The proportion of patients with history of hypertension in the increase group is higher (55% vs. 37.4%, *p* < .05). Moreover, patients in the increase group had higher respiratory rate (22 ± 5 vs. 20 ± 3, *p* < .05), lower lymphocyte (LYM) (1.13 ± 0.90 vs. 1.31 ± 0.65, *p* < .05), albumin (ALB), and globulin (GLB) level (27.07 ± 3.93 vs. 28.33 ± 4.09, *p* < .05).

To evaluate the association between delta BUN and the in-hospital mortality and other clinical outcomes in COVID-19 patients in different subgroups, we analyzed the effect of delta BUN both in patients with normal admission BUN level (<20 mg/dL) or abnormal (≥20 mg/dL) admission BUN level. Table 1 showed the in-hospital clinical outcomes of the study population, divided according to delta BUN (Increase vs. Non-increase). In-hospital mortality was significantly higher in the delta BUN increase group compared to no increase group (30.0 vs. 5.8%, *p* < .001). At the same time, in patients with normal or abnormal admission BUN, in-hospital mortality is also higher in delta BUN increase group (all *p* < .001). Moreover, patients in the delta BUN increase group also had a higher requirement of ICU admission, use of invasive mechanical ventilation and AKI (all *p* < .001).

**Table 1.** Outcomes in patients with delta BUN increase versus no increase at 24 h.

	No increase	Increase	
Delta BUN (*N* = 266)			
Transfer to Intensive care unit	28 (13.6)	23 (38.3)	*p*<.001
Invasive mechanical ventilation	12 (5.80)	18 (30.0)	*p*<.001
Acute kidney injury	7 (3.40)	13 (21.7)	*p*<.001
All-cause mortality	12 (5.80)	18 (30.0)	*p*<.001
Normal admission BUN (*N* = 217)			
Transfer to Intensive care unit	15 (8.8)	13 (27.7)	*p*<.001
Invasive mechanical ventilation	4 (2.4)	8 (17.0)	*p*<.001
Acute kidney injury	1 (0.6)	6 (12.8)	*p*<.001
All-cause mortality	4 (2.4)	8 (17.0)	*p*<.001
Abnormal admission BUN (*N* = 49)			
Transfer to Intensive care unit	12 (33.3)	11 (84.6)	*p*<.001
Invasive mechanical ventilation	8 (22.2)	10 (76.9)	*p*<.001
Acute kidney injury	5 (13,9)	8 (61.5)	*p*<.001
All-cause mortality	7 (19.4)	10 (76.9)	*p*<.001

The association between delta BUN (dichotomized as increase and non-increase) and the mortality of COVID-19 patients were further confirmed by logistic regression (Table 2). After adjusted for age, gender, hypertension, chronic obstructive pulmonary disease (COPD), respiratory rate, LYM, ALB and GLO, delta BUN during the first 24 h after admission was independently associated with the in-hospital mortality with an odds ratio (OR) of 7.427 [95% confidence interval (CI) 2.370–23.279].

**Table 2.** Odds ratios for death case associated with BUN Increase in whole population and in different subgroups.

	Model1			Model2			Model3		
	OR	(95% CI)	*p*	OR	(95% CI)	*p*	OR	(95% CI)	*p*
Total	6.391	(2.845,14.360)	<.001	5.964	(2.565,13.867)	<.001	7.427	(2.370,23.279)	.001
*Subgroups*									
Admission BUN									
<20 mg/dL	7.262	(2.027,26.018)	.002	6.475	(1.697,24.700)	.006	4.235	(1.011,17.746)	.048
≥20 mg/dL	11.667	(2.576,52.845)	.001	19.879	(3.225,122.539)	.001	111.116	(3.359.,3675.461)	.008
Hypertension									
Yes	3.750	(1.322,10.637)	.013	3.984	(1.341,11.832)	0.013	6.342	(0.890,45.209)	.065
No	10.937	(2.933,40.782)	<.001	12.179	(3.049,48.641)	<.001	12.223	(2.437,61.317)	.002

Model1: unadjusted.

Model2: adjusted for age, gender, hypertension, COPD, and respiratory rate.

Model3: adjusted for age, gender, hypertension, COPD, respiratory rate, LYM, ALB, and GLO.

OR odds ratio: CI confidence interval: COPD chronic obstructive pulmonary diseases: LYM lymphocyte: ALB albumin: GLO globulin.

The COX regression about delta BUN and the survival was summarized in Table 3. Variables with a *p* value less than .05 in univariate regression analysis were included in the multivariate regression model. In the multivariable analysis, delta BUN (increase vs. no increase) and admission BUN (<20 mg/dL vs. ≥20 mg/dL) were both associated with survival rate. BUN increase remained an independent influential factor for survival with a hazard ratio (OR) of 6.838 [95% confidence interval (CI) 3.150–14.846].

**Table 3.** Analysis by Cox regression of death during hospitalization.

Variable	HR	95%CI	*p*
*Signal-Factor Regression Analysis*			
Age, years	1.009	(0.983, 1.037)	.498
Gender, male	1.005	(0.483, 2.092)	.99
*Comorbidities*			
Hypertension	2.038	(0.957, 4.338)	.065
Diabetes mellitus	1.868	(0.799, 4.372)	.149
Coronary heart disease	1.995	(0.850, 4.680)	.112
COPD	1.585	(0.477, 5.267)	.452
* At Admission*			
Systolic blood pressure, mm Hg	1.004	(0.982, 1.031)	.6
Diastolic blood pressure, mm Hg	1.712	(0.777, 3.769)	.182
Heart rate, beats per minute	1.025	(1.001, 1.050)	.04
Respiratory rate, beats minute	1.031	(1.004, 1.058)	.025
Temperature, °C	1.613	(0.815, 3.192)	.17
O_2_ saturation, %	0.919	(0.954, 1.006)	.106
* Admission Laboratory Examination*			
Creatinine (mg/dL)	1.009	(1.005, 1.014)	*p*<.001
RBC, *1012 /L	0.834	(0.508, 1.370)	0.473
Hemoglobin, g/dL	0.994	(0.980, 1.009)	0.44
WBC, *109 /L	1.01	(0.985, 1.035)	0.434
LYM, *109 /L	0.128	(0.048, 0.343)	*p*<.001
Neutrophil, *109 /L	1.079	(1.036, 1.122)	*p*<.001
Albumin, g/L	0.836	(0.778, 0.898)	*p*<.001
Globulin, g/L	0.979	(0.891, 1.076)	.66
ALT, U/L	0.99	(0.976, 1.005)	.192
AST, U/L	0.999	(0.998, 1.000)	.166
D-dimer, ng/mL	1.138	(1.052, 1.232)	.001
CRP, mg/L	1.011	(1.006, 1.016)	*p*<.001
* Admission Coagulation Function Analysis*			
PT, s	1.031	(0.985, 1.080)	.186
APTT, s	1.027	(1.005, 1.048)	.013
TT, s	1.204	(1.102, 1.316)	*p*<.001
PLT, *109 /L	0.99	(0.985, 0.995)	*p*<.001
FIB, g/L	0.84	(0.514, 1.373)	.488
INR	1.395	(0.790, 2.466)	.251
BUN Increase in 24 h	6.838	(3.150, 14.846)	*p*<.001
BUN at admission ≥20 mg/dL	5.881	(2.719, 12.718)	*p*<.001
*Multiple-Factor Regression Analysis*			
Respiratory rate, beats minute	1.031	(1.004, 1.058)	.039
BUN Increase in 24 h	6.838	(3.150, 14.846)	.001
BUN at admission ≥20 mg/dL	5.881	(2.719, 12.718)	.017

COPD: chronic obstructive pulmonary disease; RBC: read blood cell; WBC: white blood cell; LYM: lymphocyte; ALT: alanine aminotransferase; AST: aspartate aminotransferase; CRP: C-reactive protein; PT: Prothrombin time; APTT: activated partial thromboplastin time; TT: thrombin time; PLT: platelet; FIB: fibrinogen; INR: international normalized ratio.

Overall survival in patients with different delta BUN (increase or non-increase) is presented in Figure 1. The survival curve of the increase group was significantly different from the no increase group (*p* < .001, Figure 1(A)). In pairwise comparison, patients with increase delta BUN had significant lower overall survival rate than patients with no increase delta BUN in normal admission BUN group (<20 mg/dL) and abnormal admission group (≥20 mg/dL) (Figure 1(B,C)).

**Figure 1.**
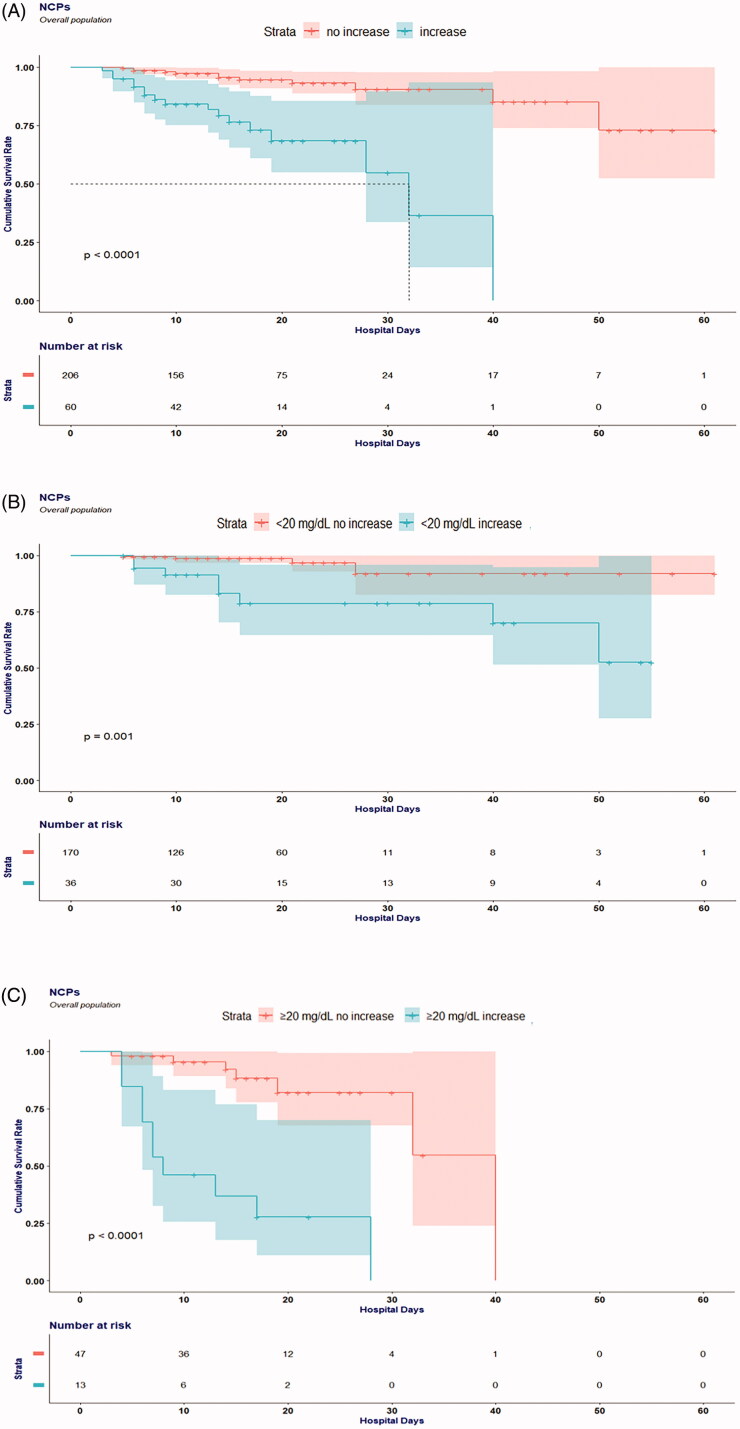
Cumulative incidence for in-hospital death of patients with coronavirus disease 2019 subgrouped by BUN value at admission and BUN change in 24 h. (A) Increase BUN versus no increase BUN at 24 h. (B) Increase BUN versus no increase BUN at 24 h when BUN at admission was normal (<20 mg/dL). (C) Increase BUN versus no increase BUN at 24 h when BUN at admission was abnormal (≥20 mg/dL).

In this retrospective study, we observed that a substantial proportion of patients (18.4%) had elevated serum BUN value at admission. However, only 16.4% patients had increased BUN 24 h after admission. We found that both elevated BUN at admission and increased BUN within first 24 h were associated with higher in-hospital mortality.

Renal injury is not uncommon in patients with COVID-19, even in those who had no underlying kidney disease [2]. Early reports found that up to 30% of patients hospitalized with COVID-19 developed AKI [3–6]. Elevated BUN at admission and increased BUN 24 h after admission may indicate early kidney injury. One possible explanation of the high prevalence of kidney involvement is the systemic immune response to the SARS-COV-2 can be detrimental in some patients, leading to so-called a cytokine storm. Besides, in kidney tissue and urine, SARS-CoV-2 RNA was also identified in COVID-19 patients [7,8]. Therefore, the kidney may be a susceptible target of this novel coronavirus.

Apart from injury of the kidney, the delta BUN may also reflect patients’ health condition. Patients with increased BUN had higher proportion of hypertension and COPD history and lower LYM, albumin and globulin level. Such patients may be functionally defective in innate and adaptive immune cell populations, making them vulnerable to COVID-19 and more likely to have worse prognosis. Therefore, monitoring BUN level might be of value. Early detection of potential or evidence AKI may facilitate appropriate treatment, including avoidance of nephrotoxic drugs and adequate fluid therapy.

In conclusion, we found that an increase in BUN at 24 h of hospitalization was associated with a composite of clinical outcomes and in-hospital mortality in patients with COVID-19. The importance of dynamically monitoring BUN may therefore be justified in the treatment of COVID-19.

## Data Availability

The data sets supporting the results of this article are included within the article. Some data used during the study are available from the corresponding author by request.
